# Djulis (*Chenopodium formosanum* Koidz.) Water Extract and Its Bioactive Components Ameliorate Dermal Damage in UVB-Irradiated Skin Models

**DOI:** 10.1155/2016/7368797

**Published:** 2016-10-26

**Authors:** Yong-Han Hong, Ya-Ling Huang, Yao-Cheng Liu, Pi-Jen Tsai

**Affiliations:** ^1^Department of Nutrition, I-Shou University, Kaohsiung, Taiwan; ^2^Department of Seafood Science, National Kaohsiung Marine University, Kaohsiung, Taiwan; ^3^Department of Food Science, National Pingtung University of Technology and Science, Pingtung, Taiwan

## Abstract

Dermal photoaging is a condition of skin suffering inappropriate ultraviolet (UV) exposure and exerts inflammation, tissue alterations, redness, swelling, and uncomfortable feelings. Djulis (*Chenopodium formosanum* Koidz.) is a cereal food and its antioxidant and pigment constituents may provide skin protection from photoaging, but it still lacks proved experiments. In this study, protective effects of djulis extract (CFE) on UVB-irradiated skin were explored. The results showed that HaCaT cells with 150 *μ*g/mL CFE treatment had higher survival and less production of interleukin- (IL-) 6, matrix metalloprotease- (MMP-) 1, and reactive oxygen species (ROS) in UVB-irradiated conditions. Subsequently, in animal studies, mice supplemented with CFE (100 mg/kg BW) were under UVB irradiation and had thinner epidermis and lower IL-6 levels in skin layer. These data demonstrate that bioactive compounds possessing the potency of antiphotoaging exist in CFE. Following that, we found rutin and chlorogenic acid (10–100 *μ*M) could significantly increase cell viability and decrease the production of IL-6 in UVB models. Additionally, djulis pigment-betanin has no effect of increasing cell viability in this study. Our findings suggest CFE can protect skin against UV-induced damage and this protection is mainly from contributions of rutin and chlorogenic acid.

## 1. Introduction

Djulis (*Chenopodium formosanum* Koidz.) is a native cereal plant in Taiwan and its nutritional values have been documented with high levels of starch, dietary fiber, proteins, and grain-limited essential amino acids (e.g., lysine). This gets djulis viewed as a whole-nutritious grain food, especially valuable for vegetarians. Apart from grain food use, djulis also gets more and more attention on the use of health food and skincare based on antioxidant and pigment-containing properties in recent years [1]. However, that still needs more experiments to clarify functional bioactivities and components of djulis.

For now, it has been known that exposure of the skin to solar UV radiation can result in reactive oxygen species- (ROS-) related oxidative stress, DNA damage, inflammatory responses, dysregulation of immune function, and even the initiation of carcinogenesis [2]. In UV lights, UVB is the main caused factor of skin photodamage, also called photoaging, due to its strong energy and permeability into skin layers. While concerning molecular mechanisms, ROS-mediated mitogen-activated protein kinase (MAPK) signaling pathway is involved and related expressions of inflammatory mediators and matrix metalloproteinases (MMPs) are often highlighted in the process. In these mediators, interleukin- (IL-) 6, TNF-*α*, and TGF*β*_1_ play crucial roles in skin abnormalities, including infiltration of inflammatory cells, redness, swelling, and uncomfortable feelings [3, 4]. As for MMPs molecules, they act as proteolytic substances which can cleavage dermal extracellular matrix such as fibrillar collagen and elastin, thus resulting in the damage of skin integrity [5]. It would be seen from this that ROS, inflammatory mediators, and MMPs could be indicators to test whether the sample possesses the ability of skin protection. For instance, through reducing the production of inflammatory cytokines, phytochemicals from* Scutellaria baicalensis* and the seeds of quinoa (*Chenopodium quinoa* Willd.) have been proved to have protective functions on skin in UVB models [6, 7]. In a classification of plant taxonomy, djulis has the same genus as quinoa. Although previous studies have pointed out bioactive compounds, including betanins, epigallocatechins, ferulic acids, and certain flavonoids, in djulis and proposed beneficial functions [1, 8], the skincare potency of djulis has never been tested under UVB models.

The aim of this study is to examine protective effects of djulis water extracts on skin photoaging in* in vitro* and* in vivo* UVB-irradiated models and confirm which bioactive compounds are main contributors.

## 2. Materials and Methods

### 2.1. Sample Preparation and Constituents Measurement

The djulis (*Chenopodium formosanum* Koidz.) seeds were harvested from the National Pingtung University of Science and Technology. The extracts were prepared by soaking 1 g of seeds grains in 5 mL of distilled water in the dark at 4°C for 24 h. After filtration, the filtrate was then freeze-dried as samples (the water extract of* Chenopodium formosanum* Koidz., CFE) for further analysis. The dry weight of this extract was 32 g and the yield was 10.6%. It was stored at −20°C until using it. To analyze components of CFE, total phenolic content was measured spectrophotometrically by using the modified Folin-Ciocalteu method, and the samples were subjected to HPLC to identify betanin and phenolic compounds according to the method described by the previous study [1].

### 2.2. Cell Culture

The keratinocyte cells from human skin (HaCaT) were grown in Dulbecco's modified Eagle medium (DMEM) supplemented with 10% (v/v) heat-inactivated fetal bovine serum, penicillin (100 units/mL), and streptomycin (100 units/mL). These culture reagents were commercial products (Gibco, Grand Island, NY, USA). The cells were incubated in an atmosphere of 5% CO_2_ at 37°C and were regularly subcultured for experiment uses. In samples test, cells were seeded in a 96-well plate at a concentration of 1 × 10^5^ cells/mL. Following one-day incubation, cells media were removed and replaced by phosphate buffer saline (PBS) for UV irradiation. The plate was placed in an UV CROSSLINKER (XLE-1000B, SPECTROLINE), which set peak spectral emission, electric tension, frequency, and the distance from cells at 315 nm, 120 V, 60 Hz, and 15 cm, respectively. When cells were exposed to 30 mJ/cm^2^  UVB, time of irradiation was averagely 30 sec. Then, these cells were treated with media containing various concentrations of CFE, pure compounds from CFE, or ascorbic acid for 24 h. After that, cell supernatants were collected for the analysis of inflammation-related mediators and cells in the bottom of plate were for cell viability.

### 2.3. Cell Viability

The cell viability was assayed by MTT (3-(4,5-dimethylthiazol-2-yl)-2,5-diphenyltetrazolium bromide) method. After collecting cell supernatants, an aliquot of 55 *μ*L/well of DMEM containing 0.5 mg/mL MTT was added into the plate for 3 h incubation. Then, the plate was added with an aliquot of 100 *μ*L/well of isopropanol containing 0.04 N HCl and appropriately shaken for dissolving out colored crystals. Cell viability values at absorbance of 570 nm were determined by an ELISA reader (Bio-Rad Laboratories, Hercules, CA, USA). In this study, to confirm the data of cell viability, we counted cell number by trypan blue staining. Briefly, we seeded cells onto 6 cm dish (1 × 10^6^ cells) for 24 h incubation. After washing twice by PBS, cells were irradiated with UVB at dose of 30 mJ/cm^2^. Then, these cells were treated with CFE for 24 h. Following that, cells were harvested for counting cell number.

### 2.4. IL-6 and MMP-1 Measurements

Production of IL-6 (eBioscience, Minneapolis, MN, USA) and MMP-1 (R&D, Minneapolis, MN, USA) in cell supernatants was assayed using commercial ELISA kits. Briefly, primary anti-IL-6 or MMP-1 antibodies were coated onto 96-well plates. After overnight incubation, plate wells were washed with washing buffer and blocked with blocking solution for 1 h. After washing wells, diluted supernatants or standard reagents were added into wells and the plate was incubated for 2 h. Next, wells were washed and then added with biotin-conjugated anti-IL-6 or MMP-1 antibodies for 1 h. Following the wash, the wells were added with horseradish peroxidase- (HRP-) conjugated streptavidin for 30 min, washed, and incubated with tetramethylbenzidine. Absorbance was measured by an ELISA reader at 620 nm. Data were calculated according to standard curves.

### 2.5. Intracellular Reactive Oxygen Species (ROS) Measurement

Detecting levels of dichlorofluorescein (DCF) which is derived from dichlorodihydrofluorescein-diacetate (DCFDA) under oxidative stress can represent the production of ROS. In this experiment, cells were seeded at a concentration of 1 × 10^6^ cells in a 6 cm dish for 24 h and irradiated with UVB (30 mJ/cm^2^); then, they were treated with CFE or positive controls for 3 h. Next, cells were collected in a tube and labeled with 5 *μ*M DCFDA (Invitrogen, Carlsbad, CA, USA) for 30 min. After that, cells were centrifuged and washed twice with phosphate buffer saline (PBS) and intracellular DCF levels were detected by flow cytometer (Accuri Cytometers, Inc., Ann Arbor, MI, USA).

### 2.6. Cell Cycle Analysis

Cell cycle analysis was performed to determine the proportion of apoptotic sub-G_1_ hypodiploid cells. In brief, cells were seeded at a concentration of 1 × 10^6^ cells in a 6 cm dish for 24 h. Then, cells were irradiated with UVB (30 mJ/cm^2^) and treated with CFE or positive controls for 24 h. Afterwards, cells were harvested and fixed in 70% ethanol for 24 h at −20°C; then, they were washed twice with PBS and introduced with PBS containing PI solution at 4°C. Following overnight incubation, cellular fluorescein levels were assayed by flow cytometer. In the assay, each cycle phase was quantified from histograms by using software programs and changes of values in cycle phases were calculated to evaluate CFE's effects.

### 2.7. Animals Study

Eight-week-old female BALB/c mice were purchased from the National Animal Center (Taipei, Taiwan). Mice were maintained in an air-conditioned room at 23 ± 2°C on a regulated 12 h light-dark cycle and fed a nonpurified diet (Lab Rodent Chow 5001, Ralston Purina Inc., St. Louis, MO, USA) for adaptation. Animal care and handling are conformed to the National Institutes of Health's Guide for the Care and Use of Laboratory Animals. In oral experiment, mice at age of 10 wk were divided into five groups, control, vehicle, LCFE (40 mg/kg BW), HCFE (100 mg/kg BW), and AA (ascorbic acid, 25 mg/kg BW), and each group had 8–10 mice. Water or samples were orally administered to these mice for 14 consecutive days. On day 8, all mice dorsal skin hairs were removed by medical shaver, and they received UVB irradiation (150 mJ/cm^2^) for 7 consecutive days except for the control mice. Mice skin thickness was daily measured once by using a Quick Mini caliper (Verniercaliper, number 1202-100, Beijing C & C Trad., Beijing, China) until mice sacrifice. Following the sacrifice, mice dorsal and ear skin tissues were collected for histology observation. In topical experiment, forty mice were averagely assigned into four groups: control (emulsion reagent), vehicle (emulsion reagent), CFE (8 mg/mouse in emulsion reagent), and AA (0.392 mg/mouse in emulsion reagent). After all mice dorsal skin hairs were removed, mice received 150 mJ/cm^2^ UVB irradiation for seven consecutive days. The control mice did not receive UVB irradiation as shame mice. In each day, mice received topical application with respective samples at volume of 200 *μ*L after UVB irradiation. Then, mice skin thicknesses were daily measured until mice sacrifice. After sacrifice, mice dorsal and ear skin tissues were collected for epidermis histology observation.

### 2.8. Tissue Histology

Skin specimens were fixed in 10% buffered formalin. Tissue sections measuring 3 *μ*m were created from the paraffinized blocks and stained with hematoxylin and eosin (TA01HE kit, BioTnA Inc., Kaohsiung, Taiwan). For further immunohistochemistry, the paraffin sections were deparaffinized in xylene and rehydrated in a graded alcohol series, treated with 3% H_2_O_2_ for 10 min, and boiled with the citrate buffer (pH 6) for 40 min, then blocking with the immunoblock for 1 h at room temperature. The sections were incubated with rabbit anti-IL-6 antibody (bs-0379R, Bioss Inc., Woburn, MA, USA) or rabbit anti-collagen I antibody (ab34710, Abcam Inc., Cambridge, MA, USA) for 2 h and then treated with mouse/rabbit probe HRP labeling (BioTnA, TAHC03D) for 30 min at room temperature. Peroxidase activity was developed in diaminobenzidine-H_2_O_2_ solution (BioTnA, TAHC03D) for 5 min at room temperature and counterstain with hematoxylin (BioTnA, TA01NB).

### 2.9. Statistical Analysis

All data are presented as means ± SD or SEM of at least three independent experiments. Significant differences among the groups were determined by using unpaired Student's* t*-test and Duncan's multiple range tests. During UV irradiation period, skin thicknesses among groups were statistically analyzed and compared by using the linear mixed model (LMM). *p* value less than 0.05 was considered statistically significant.

## 3. Results and Discussions

### 3.1. CFE Protects from UVB-Induced Cell Deaths in Cell Models

**Figure 1:**
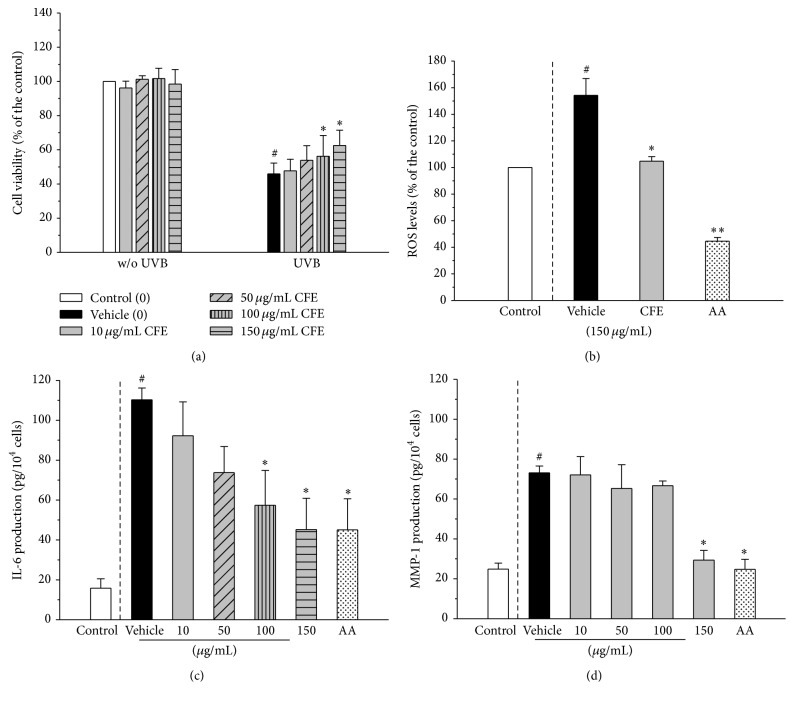
Effects of CFE treatments on cell viability (a) and the production of ROS (b), IL-6 (c), and MMP-1 (d) in UVB-irradiated HaCaT cells. Ascorbic acid (AA) at concentration of 50 *μ*M was as a positive control. Compared with the vehicle, the statistical difference was analyzed by Student's* t*-test and indicated a significance as ^*∗*^ *p* < 0.05 or ^*∗∗*^ *p* < 0.01. Compared with the control, the statistical difference was analyzed indicated a significance as ^#^ *p* < 0.05.

This study used keratinocyte cell line exposed to UVB radiation to mimic the model of skin photoaging. As cells were under UVB irradiation at doses of 0–100 mJ/cm^2^, we found UVB at dose of 20–30 mJ/cm^2^ caused cells mortality of 40–50%. Previous studies have indicated that UVB at dose of 20–40 mJ/cm^2^ is close to the average minimal erythema dose (MED) for human populations [9, 10]. Thus, UVB at dose of 30 mJ/cm^2^ was conducted in the following experiments. In Figure 1(a), CFE at concentrations of 10–150 *μ*g/mL did not cause HaCaT cells deaths (without UVB irradiation). While cells were under UVB exposure, their viability decreased by 60%; however, the decrease could be reversed by CFE treatments (Figure 1(a) right). We confirmed the effect of CFE on cell viability by using trypan blue staining. The data showed that CFE (150 *μ*g/mL) could have higher cell number compared to the vehicle in an UVB-irradiated model (CFE, 0.84 ± 0.09 × 10^6^ cells, versus vehicle, 0.65 ± 0.10 × 10^6^ cells). In addition, the observation in Table 1 that 30% of UVB-irradiated cells (vehicle) were arrested in Sub-G_1_ phase (a DNA content less than 2 n) was given, whereas cells with CFE treatment at concentration of 150 *μ*g/mL only has 13.5 ± 5.9% arrest in Sub-G_1_ phase and higher S + G_2_/M phase. It is also known that UVB-induced cell death is mainly through apoptosis [11]. Further, it is essential to confirm if CFE might alter DNA intactness and cause precancerous DNA-damaged cells to survive. We detected the degree of DNA methylation by a commercial kit (ab117128, Abcam) as the assay of DNA protection. The data show there was no change of percentage of 5-methylcytosine (5-mC) in DNA in cells with UVB irradiation (30 mJ/cm^2^) compared to the control (1.87% versus 1.71%). Meanwhile, CFE had comparable percentage of 5 mC in cells (1.79%), showing CFE did not affect DNA intactness (unpublished data). The data indicate that CFE might attenuate cell apoptosis and subsequently augment cell viability in UVB-irradiated keratinocytes.

### 3.2. CFE Reverses UVB-Induced Cellular Mediators in Cell Models

Oxidative stress and mediators derived from UVB irradiation are the main cause of dermal harmful effects, including inflammation, structural alteration, and even more cells deaths. Therefore, we assayed the production of reactive oxygen species (ROS), IL-6, and MMP-1. As shown in Figure 1(b), HaCaT cells under UVB radiation (vehicle) would have 1.6-fold ROS production compared to the control, whereas CFE treatment at concentration of 150 *μ*g/mL made UVB-irradiated cells release less ROS production that is comparable to that of the control. It is well known that UV radiation causes ROS-mediated oxidative stress which triggers cellular transcriptional activations, including NF-*κ*B, AP-1, and MAPK induction or STAT3 tyrosine phosphorylation, increasing the production of inflammatory mediators and progressive cell death [12, 13]. Accordingly, scavenging ROS is expected to regulate skin photodamage. Our previous studies show CFE is a strong antioxidant to remove free radicals [1, 14]; hence we suggest that CFE's potency results from its antioxidant.

**Figure 2:**
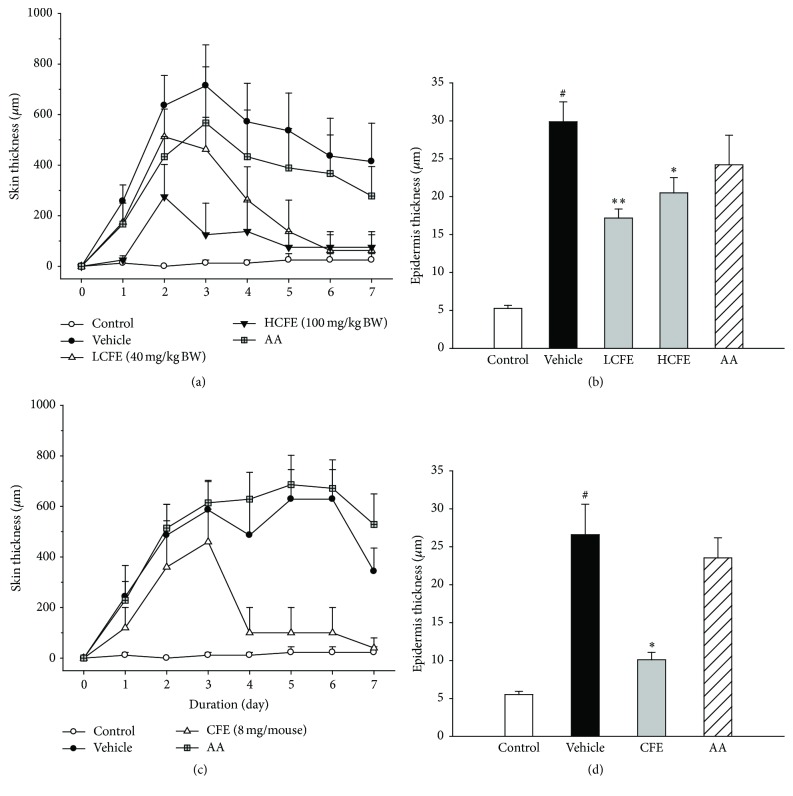
Effects of CFE treatments on skin (a, c) and epidermis (b, d) thickness in UVB-irradiated mice. (a), (b) and (c), (d) show the data of oral and topical application experiments, respectively. Bar values are means ± SEM, *n* = 8–10. Compared with the vehicle, the statistical difference was analyzed by Student's* t*-test and indicated a significance as ^*∗*^ *p* < 0.05 or ^*∗∗*^ *p* < 0.01. Compared with the control, the statistical difference was analyzed and indicated a significance as ^#^ *p* < 0.05.

For the change of cellular response, UVB-induced production of IL-6 and MMP-1 was significantly reduced by CFE treatments, dosedependently (Figures 2(c) and 2(d)). In this experiment, 50 *μ*M ascorbic acid (AA) was used as the positive control to confirm our data, and AA did have the dermal protection. The same tendency is adherent to a Boyce et al.'s study [15].

### 3.3. CFE Improves UVB-Induced Skin Alterations in Animal Models

**Figure 3:**
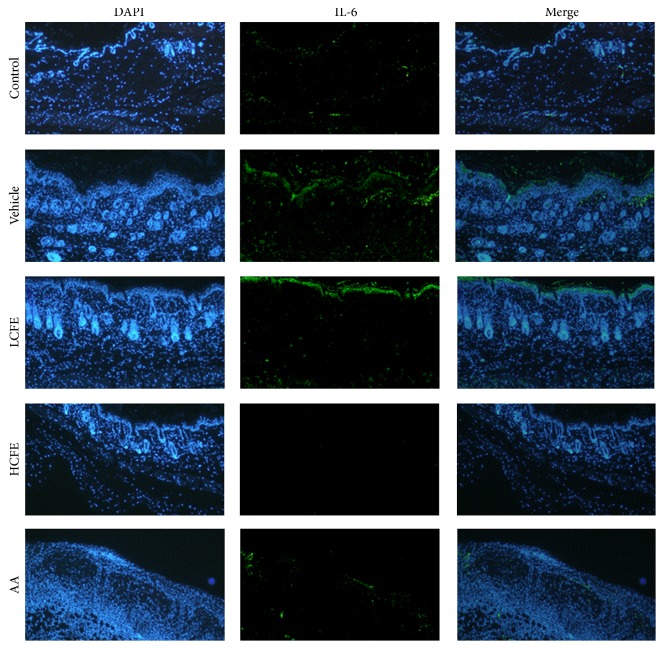
Effects of CFE treatments on skin IL-6 production in UVB-irradiated mice. In experiments, mice skin biopsies were processed for staining. A fluorescent microscopic image (100x) showing IL-6 stained mouse skin cells in green and 4′,6-diamidino-2-phenylindole (DAPI) stained all nuclei in blue (100x).

Subsequently, we conducted animal experiments to prove* in vivo* effects of CFE. In Figure 2(a), the vehicle (only UVB-irradiated) mice's skin thickness increased day by day (*p* < 0.001) and had a closely 700 *μ*m increase at day 3 compared to the control. But this increase was significantly attenuated by CFE supplement at doses of 40 and 100 mg/kg BW. For the measurement of epidermis changes, collected mice dorsal skins were stained with H&E. The data in Figure 2(b) revealed that mean epidermis thickness in CFE supplement mice was significantly less than that in the vehicle mice. Furthermore, we explored whether skin protection could be displayed via topical application of CFE. As shown in Figure 2(c), emulsion CFE could reduce the increased skin thickness compared to other groups during UVB radiation period (all *p* values < 0.05 by LMM model). The same event was represented in tissues staining (Figure 2(d)), and CFE treatment (10.1 ± 0.96 *μ*m) had less epidermis thickness than the vehicle's (26.6 ± 4.02 *μ*m). This data shows that CFE intervention by oral or skin administration has the beneficial protection from UVB-induced skin alterations. To investigate the modulation of cellular mediators by CFE treatment in animal models, we used IHC assay to detect the expressions of IL-6 and collagen in skin. As shown in Figure 3, compared to the vehicle group, dermal IL-6 expression in the HCFE group was significantly decreased. Dermal collagen type I expressions in CFE groups were slightly higher than that in the vehicle group (data not shown).

When the skin is under UVB radiation, proinflammatory cytokines would be strongly produced to cause cutaneous inflammation and their outcomes including erythema, edema, and epidermal hyperplasia are often recognized as markers of skin damage [16, 17]. In our test, UVB did induce significantly the production of IL-6 that is close to 150–200 pg/10^4^ cells in cell culture tests, and the same tendency is also seen in animal models (Figure 3). Since IL-6 is involved in the regulation of MAPK signaling cascade and stimulates the production of MMPs which can cause fibrillar collagen breakdown and thus leads to premature skin damage [18, 19], it is suggested that CFE can lower IL-6 and make less dermal structural alterations in here.

Besides, improvement effects of CFE by oral route can work in UVB models, making us have an interest in exploring the linkage between skin and systemic immune. We analyzed splenocytic cytokines profile of mice in this study and found IL-6 production in the vehicle mice is 2.5-fold that in the control, whereas mice with CFE supplement had lower production (the vehicle versus HCFE, 1.98 ± 0.39 versus 0.74 ± 0.11 ng/mL; *p* = 0.02). Thus, we suggest that CFE supplement potentially modulates systemic inflammation and T cell function, which might be correlated with the improvement of skin alterations. This also demonstrates that IL-6 is a key factor in protection against UVB-induced skin alterations.

### 3.4. Main Phytochemicals Contribute to CFE's Beneficial Potency in UVB-Irradiated Models

**Figure 4:**
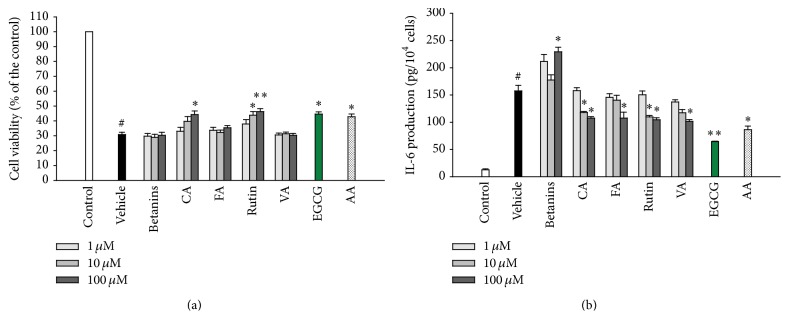
Effects of identified compounds from CFE on cell viability (a) and IL-6 production (b) in UVB-irradiated HaCaT cells. Betanins, chlorogenic acid (CA), ferulic acid (FA), rutin, vanillic acid (VA), EGCG (20 *μ*M), and ascorbic acid (AA, 50 *μ*M) were used. Bar values are means ± SEM, *n* = 5. Compared with the vehicle, statistical differences were analyzed by Student's* t*-test and indicated a significance as ^*∗*^ *p* < 0.05 or ^*∗∗*^ *p* < 0.01. The statistical difference between the control and vehicle represents a significance as ^#^ *p* < 0.05.

In CFE, this study identified eight phytochemical compounds, including betanin (1643 mg/100 g), rutin (330 mg/100 g), vanillic acid (85 mg/100 g), chlorogenic acid (65 mg/100 g), gallic acid (51 mg/100 g), epicatechin (43 mg/100 g), and ferulic acid (9 mg/100 g). The ration of compounds content showed betanin was the major pigment and rutin was the major constitute of polyphenols, followed by chlorogenic acid and catechins. To analyze the effectiveness of compounds (Figure 4), we found rutin (10 *μ*M) and chlorogenic acid (100 *μ*M) could significantly increase cell viability but decrease the production of IL-6. The data imply these two are possible active components of CFE in UVB models. For rutin, its protection from cell death of HaCaT cells under chronic UVC exposure (1.8 J/cm^2^) and inhibition in MMP-1 production from dermal fibroblasts under UVB exposure had been reported [20, 21]. Basically, our data is attached to these reports. For chlorogenic acid, no studies clearly proved its effects in UVB models; only a report supposed its antioxidant and photostability to UV light could fight against UV irradiation [22]. In here, we experimentally proved chlorogenic acid had the protective potency.

Besides, treatment of vanillic acid or ferulic acid (100 *μ*M) did not increase cell viability but decreased the production of IL-6 (Figure 4(b)). These two components have been indicated as major improved contributors of* Chromolaena odorata* extracts in H_2_O_2_- and xanthine oxidase-induced models [23]. This might demonstrate that these two also provide a little part of anti-inflammation in CFE. Out of our expectation, reverse data had been seen; djulis pigment-betanin (100 *μ*M) in this model did not affect cell viability but increased IL-6 production. Although this dose is 20-fold the converted dose of betanin (~4.5 *μ*M) driven from CFE (150 *μ*g/mL), betanin at dose manner is notable to discuss its different effects in future work. In this experiment, the positive control, EGCG, and ascorbic acid act as positive controls, in reference to previous studies [2, 24].

## 4. Conclusion

Our findings show that CFE can provide protection from UVB-induced skin damage, and rutin and chlorogenic acid in CFE act as main bioactive compounds in skin protection.

## Figures and Tables

**Table 1:** The effect of CFE treatment on cell cycle in UVB-irradiated HaCaT cells.

	UVB 30 mJ/cm^2^	% of cells			
		Sub-G_1_	G_0_/G_1_	S	G_2_/M
Control	−	3.7 ± 1.8	32.0 ± 10.8	15.8 ± 5.3	28.8 ± 6.0
Vehicle	+	29.7 ± 7.0^#^	28.1 ± 5.5	15.5 ± 4.7	14.3 ± 5.2^#^
CFE (150 *μ*g/mL)	+	13.6 ± 5.9^*∗∗*^	31.3 ± 2.4	14.3 ± 2.6	22.9 ± 3.1^*∗∗*^
AA (20 *μ*M)	+	5.5 ± 0.7^*∗∗*^	43.0 ± 5.5^*∗∗*^	22.6 ± 4.2^*∗*^	20.7 ± 3.6^*∗*^

Each value represents the mean ± SD of three independent experiments. Compared with the vehicle, the statistical difference was analyzed by Student's *t*-test and indicated a significance as ^*∗*^ *p* < 0.05 and ^*∗∗*^ *p* < 0.01. Compared with the control, the statistical difference was analyzed and indicated a significance as ^#^ *p* < 0.05.
